# Drainage of the Ventricles in Meningitis

**Published:** 1892-06-04

**Authors:** 


### SURGERY.

### DRAINAGE OF THE VENTRICLES IN MENINGITIS.

Dr. Wynter has recorded four cases of tubercular meningitis treated by draining the subarachnoid space of the cord.* Mr. Kendal Franks† records a case of tubercular meningitis in which temporo-sphenoidal abscess was sus-

* *Lancet*, 91, Vol. 1. † *British Medical Journal* December 6, '90, p. 1,294.

pected, and he trephined the temporo-sphenoidal lobe, and although he did not find an abscess, the lateral ventricle was punctured and drained by a small tube passed through the convolutions. The child was comatose and hemiplegic before the operation, but consciousness returned after, and the hemiplegia lessened, but although drainage was maintained (the post-mortem showed no excess of fluid), the coma soon returned, and the hemiplegia increased. The post-mortem showed extensive tubercular meningitis.

In the cases recorded by Dr. Wynter, the results were not as good even as in Mr. Kendal Franks' case. In one there was slight improvement in the general condition, and in another contraction of unduly dilated pupils, and in the other two no change. But drainage was not maintained in all the cases.

Mr. Mayo Robson* records a case of possible meningitis from ear disease, with hemiplegia, or aphasia, in which he trephined for abscess, but did not find one, but drew off six ounces of clear fluid from the lateral ventricle. The dura mater was sutured. Power returned in the arm next day, and soon after in the leg, and aphasia began to pass off on the third day, and the child recovered. We cannot help feeling very doubtful whether in this case the operation had anything to do with the child's recovery, as the fluid would have been very quickly re-secreted and no drainage was used; and the relief was not rapid enough to make one think it could have been due to the relief of pressure.

In a paper† read at the las annual meeting of the British Medical Association, Mr. Morton discusses the probability of our being able to relieve cases of tubercular meningitis by tapping the subarachnoid space of the cord. He shows that the increased pressure in the ventricles, probably, at any rate, helps to cause the softening around, and that there is some relation between the amount of softening and the duration of the coma. He also contrasts some of the symptoms of the late stage of tubercular meningitis with those of surgical compression, and points out how impossible it is to put down such unilateral symptoms as we sometimes get in the late stage of tubercular meningitis, such as hemiplegia, conjugate deviation of the eyes, &c., to general increase of pressure in the ventricles. Altogether there seems little *a priori* evidence in favour of this procedure, and Mr. Kendal Franks' case we consider most discouraging. Granting that the drainage relieved, yet the child speedily died. Mr. Franks says: "The cause of death was apparent on removing the brain, for its base was found to be the seat of tuberculous purulent meningitis." But that is just what we might expect in such a case, and the question is, Given this condition, will draining the ventricles do any good? This case gives us no encouragement at any rate, for with intra-ventricular pressure completoly relieved, yet the child dies from his meningitis all the same.

Mr. Mayo Robson says if we can do good by draining the peritoneum in tubercular peritonitis, we ought to be able to cure tubercular meningitis by tapping the ventricles, and he gives hopes we may. But we have surely very different conditions to deal with, and no analogy to guide us. It is not the pressure of the fluid which kills in tubercular peritonitis; the removal of the fluid is undertaken now generally to cure the local tuberculosis. In the case of tubercular meningitis, it is not proposed to cure the tubercle, but to relieve pressure in an acute condition; and it seems to us that it has yet to be shown that death is due to this pressure. The condition is certainly analogous to tapping a pleural effusion to relieve dyspnœa from pressure on the lung and displacement of the heart, but here we feel sure of the cause of the symptom which is so serious, but we cannot say the coma, and failing cardio respiratory action are certainly due to increased intra ventricular pressure in meningitis.

* *B. Med. Journal*, Dec. 6th, 1892. † *B. Med. Journal*, Oct. 17th, 1891.

It is quite possible, in Mr. Kendal Franks' case, the brain may have been so softened around the ventricles, as it often is in tubercular meningitis, that the damage done by the pressure could not be repaired, and so that we ought to tap quite early in these cases before they become comatose. A lung can re-expand, and a displaced heart can return, but a brain softened by pressure may be too spoiled to take on its functions again. And yet, in Kendal Franks' case, it did for a time; or, at any rate, coma passed off. But the parts that get softened in tubercular meningitis—the corpus callosum and the great basal ganglia—are not the parts a lesion of which one would expect to cause coma or cardio-respiratory failure. We do not find the convolutions or the medulla softened in these cases. Kendal Franks' case, then, proves that it is not merely the effect of pressure on the convolutions or medulla which causes death, or his patient would have recovered; and, on the other hand, these parts are not softened, as are the basal ganglia and corpus callosum, with the white matter immediately around the lateral ventricles.

Another point raised by Dr. Wynter and Mr. Robson's and Mr. Frank's cases, is whether by draining the subarachnoid space of the cord we can also drain the ventricles, and whether, if so, this or direct drainage through the skull, is the best. We are certain from repeated careful examination of the opening from the subarachnoid space of the cord into the fourth ventricle—the so-called foramen of Majendie—which is really not a foramen at all—that it is not blocked in tubercular meningitis, and that, therefore, drainage of the subarachnoid space of the cord will drain the ventricles, and we cannot help thinking that very slow drainage through a fine trochar or tube left in the subarachnoid space of the cord after laminectomy in the upper lumbar region, is less likely to do harm than draining continuously right through the cerebral hemisphere.

Are we justified in trying to relieve these cases in this way? They are practically hopeless; slight improvement seems to have resulted in two or three cases. If we wait until coma comes on, no anæsthetic is required, but we think if we are going to drain at all we had better not wait for coma in case too much damage is done by that time by the production of softening around. We could never press such a last and slender hope as this proceeding seems to be. Having regard to the desperate condition of the patient we might try it in a few more cases.

